# NTCP Models for Severe Radiation Induced Dermatitis After IMRT or Proton Therapy for Thoracic Cancer Patients

**DOI:** 10.3389/fonc.2020.00344

**Published:** 2020-03-17

**Authors:** Giuseppe Palma, Serena Monti, Manuel Conson, Ting Xu, Stephen Hahn, Marco Durante, Radhe Mohan, Zhongxing Liao, Laura Cella

**Affiliations:** ^1^Institute of Biostructures and Bioimaging, National Research Council, Naples, Italy; ^2^National Institute for Nuclear Physics, (INFN), Naples, Italy; ^3^Department of Advanced Biomedical Sciences, Federico II University School of Medicine, Naples, Italy; ^4^Department of Radiation Oncology, University of Texas MD Anderson Cancer Center, Houston, TX, United States; ^5^GSI Helmholtz Centre for Heavy Ion Research, Department of Biophysics, Darmstadt, Germany; ^6^Department of Radiation Physics, University of Texas MD Anderson Cancer Center, Houston, TX, United States

**Keywords:** radiation dermatitis, dose-surface histogram, proton therapy, intensity modulated radiation therapy, NSCLC, NTCP

## Abstract

Radiation therapy (RT) of thoracic cancers may cause severe radiation dermatitis (RD), which impacts on the quality of a patient's life. Aim of this study was to analyze the incidence of acute RD and develop normal tissue complication probability (NTCP) models for severe RD in thoracic cancer patients treated with Intensity-Modulated RT (IMRT) or Passive Scattering Proton Therapy (PSPT). We analyzed 166 Non-Small-Cell Lung Cancer (NSCLC) patients prospectively treated at a single institution with IMRT (103 patients) or PSPT (63 patients). All patients were treated to a prescribed dose of 60 to 74 Gy in conventional daily fractionation with concurrent chemotherapy. RD was scored according to CTCAE v3 scoring system. For each patient, the epidermis structure (skin) was automatically defined by an in house developed segmentation algorithm. The absolute dose-surface histogram (DSH) of the skin were extracted and normalized using the Body Surface Area (BSA) index as scaling factor. Patient and treatment-related characteristics were analyzed. The Lyman-Kutcher-Burman (LKB) NTCP model recast for DSH and the multivariable logistic model were adopted. Models were internally validated by Leave-One-Out method. Model performance was evaluated by the area under the receiver operator characteristic curve, and calibration plot parameters. Fifteen of 166 (9%) patients developed severe dermatitis (grade 3). RT technique did not impact RD incidence. Total gross tumor volume (GTV) size was the only non dosimetric variable significantly correlated with severe RD (*p* = 0.027). Multivariable logistic modeling resulted in a single variable model including *S*_20Gy_, the relative skin surface receiving more than 20 Gy (OR = 31.4). The cut off for *S*_20Gy_ was 1.1% of the BSA. LKB model parameters were TD_50_ = 9.5 Gy, *m* = 0.24, *n* = 0.62. Both NTCP models showed comparably high prediction and calibration performances. Despite skin toxicity has long been considered a potential limiting factor in the clinical use of PSPT, no significant differences in RD incidence was found between RT modalities. Once externally validated, the availability of NTCP models for prediction of severe RD may advance treatment planning optimization.

## Introduction

The development of acute and chronic radiation-induced skin injuries is a common side effect of radiation therapy (RT). Acute radiation dermatitis (RD), with reactions evident one to four weeks after the beginning of RT, may limit the duration of treatment and the dose delivered (1, 2). The severity of adverse dermatologic events ranges from mild erythema to moist desquamation and ulceration, impacting on the quality of a patient's life (3). Acute RD occurs most frequently after RT of breast, pelvic (e.g., anal cancer, vulvar cancer) and head and neck malignancies, while lower incidence is reported for deeper tumors as lung cancers (4).

Thanks to the advent of high-energy photon RT, which provide more skin sparing treatments compared to older ones with lower energy treatment machines, a general reduction in RD incidence and severity has been achieved in the past decades. Still, RD remains one of the significant adverse effect of RT.

The introduction of most modern treatment modalities, such as intensity modulated RT (IMRT) or proton beam therapy, has nowadays changed the dose distribution patterns in the normal tissues surrounding the tumors (5, 6). Accordingly, advanced RT techniques have generally reduced the burden of radiation related risks, included skin toxicity (7, 8). The substantial sparing of organs-at-risk from proton beams compared to IMRT is expected to theoretically further reduce radiation-induced morbidity (9). However, the risk of a potential increase of skin toxicity has long been considered a peculiar drawback in the clinical use of protons. The higher beam entry dose of the spread-out Bragg peak represents a disadvantage for the skin; thus causing concern over a possible increase in skin adverse effects (10, 11).

The skin response to radiation has been studied since the discovery of X-rays (2, 12). Multiple patient-specific and dosimetric features have been identified as risk factors for acute skin toxicity after RT for diverse tumor locations, in particular breast (7, 13, 14), head and neck (15) or brain tumors (16). Notwithstanding this, normal tissue complication probability (NTCP) modeling of skin toxicity is still not fully explored. In addition, the available NTCP models are mostly designed for dose-volume histogram (DVH) from a target volume (e.g., breast) (17–20) or are based on DVH from a pseudo-skin structure defined as a layer of 2-5 mm inward from the body contour (15, 21, 22). A different approach could directly consider the surface phenomena connected to the actual organ at risk, i.e., the skin (23).

In the present study, we analyzed the incidence of acute RD in thoracic cancer patients treated with Intensity-Modulated RT (IMRT) or Passive Scattering Proton Therapy (PSPT) on a completed prospective randomized trial (24), and we developed NTCP models for severe acute RD. The model procedure was based on the introduction of a fully automated method for skin definition as a critical organ. Both the Lyman-Kutcher-Burman (LKB) and multivariable logistic regression modeling strategies were adopted.

## Methods and Material

The study involved 225 patients with locally advanced Non-Small-Cell Lung Cancer (NSCLC) enrolled in the trial NCT00915005. One hundred sixty-six patients were eligible for the present analysis. The eligibility criteria included acute RD follow-up data and availability of dose maps. All patients were treated according to an IRB approved protocol (NCT00915005) with image-guided IMRT (103 patients) or PSPT (63 patients) to a prescribed dose of 66 or 74 Gy (RBE) in 33 or 37 conventional daily fractions delivered with concurrent chemotherapy (CHT). The typical three-field arrangement was used for all PSPT plans (24). Typically, a posterior and lateral beams plus an oblique beam that avoids lung parenchyma in its exit dose (25). In the IMRT plans, six to nine equidistant, coplanar, axial 6-MV beams were usually used (26).

Details of the protocol, patient and treatment characteristics are reported elsewhere (27, 28). All dose maps were obtained with a dose grid size of 2.0 × 2.0 × 2.5 mm^3^.

For each patient, acute RD was assessed as the maximum score recorded during the treatment and within 90 days after RT. The RD was graded according to the National Cancer Institute's Common Toxicity Criteria for Adverse Events (CTCAE) version 3 into the following groups:

*Grade 1*: Faint erythema or dry desquamation
*Grade 2*: Moderate to brisk erythema; patchy moist desquamation, mostly confined to skin folds and creases; moderate edema
*Grade 3*: Moist desquamation in areas other than skin folds and creases; bleeding induced by minor trauma or abrasion
*Grade 4*: Life-threatening consequences; skin necrosis or ulceration of full thickness dermis; spontaneous bleeding from involved site; skin graft indicated.

### Dosimetric Analysis

For each patient, individual DICOM RT plans (computed tomography (CT) scans, doses, and contoured organ structures) were converted into Matlab-readable format (MathWorks, Natick, MA, USA) using the CERR (Computational Environment for Radiotherapy Research) software (29).

**Figure 1:**
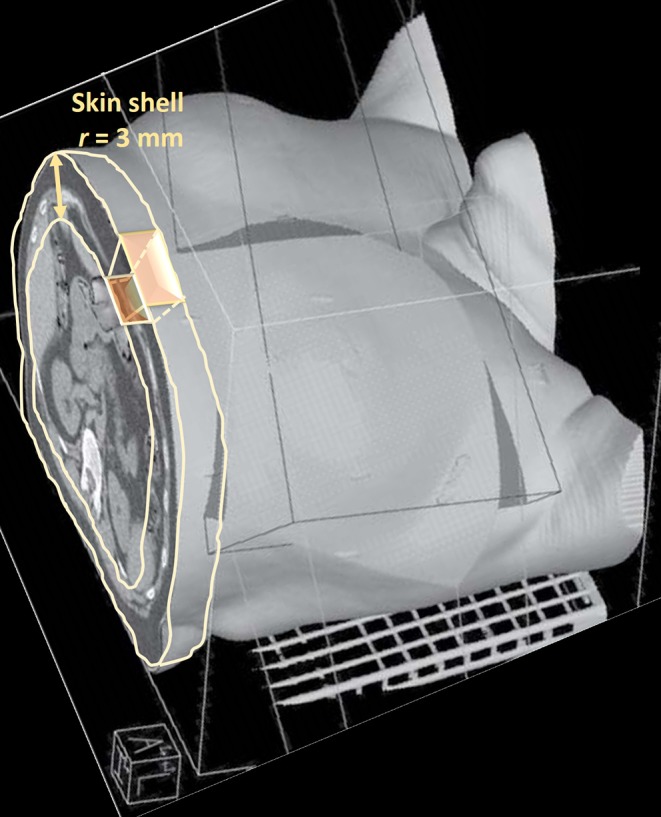
Pictorial representation of the skin segmentation and dose-surface histogram extraction.

The epidermis (skin) was automatically defined by an in-house segmentation algorithm developed on purpose. In detail, the body contour was first corrected applying a Hounsfield unit thresholding over a moving window to exclude possible contribution from treatment bed. The resulting structure Ω was then eroded by 3 mm [i.e., approximately the mean skin thickness (30)]; the skin was then obtained subtracting from Ω its erosion (Figure 1) according to the following equation

$$\begin{array}{l} {\text{skin}}_{r}=\left[\Omega \backslash \;\left(\Omega ⊝B\left[r\right]\right)\right]\\ \text{ skin}={\text{skin}}_{3\text{ mm}} \end{array}$$

where *B*[*r*] is a spherical structuring element of radius *r*, \ represents the set difference, and ⊝ stands for morphological erosion (31).

The absolute dose-surface histograms (DSHs) of the skin thus extracted were computed by an in-house developed library for Matlab (23) according to

$$\text{DSH}\left(x\right)=\underset{r\to 0}{\text{lim}}\frac{\text{DVH}{\left(X\right)}_{{\text{skin}}_{r}}}{r}$$

The relative DSH were obtained using the Body Surface Area (BSA) index as scaling factor. The BSA index was calculated according to

$$\text{BSA}=7.184.{\text{cm}}^{2}{\left(W/\text{kg}\right)}^{0.425}.{\left(H/\text{cm}\right)}^{0.725}$$

Where *W* and *H* are patient's weight and height respectively (32).

The following DSH metrics were extracted: the relative skin surface receiving more than *X* Gy (*S*_*x*_) in step of 1 Gy, the minimum dose given to the hottest *x*% skin surface in step of 5% (*D*_*x*_), the skin near maximum dose (*D*_2%_) and the mean dose (*D*_mean_).

### Statistical Analysis

Acute RD was analyzed according to its severity, i.e., grade 3 (G3) RD vs. G0-G2 RD. All the extracted skin dose parameters along with patient-specific and treatment-related factors were analyzed by univariate statistical methods for the above defined grouping. Categorical variables were tested by Pearson's χ^2^-test or Fisher's exact test when appropriate; continuous variables were tested by Mann-Whitney *U*-test.

Average relative DSHs stratified by treatment modality and toxicity endpoints were compared at each dose point by two-tailed *t*-test. A significance α-level of 0.05 corrected according to the Holm–Šidák method for multiple comparison was applied (33).

### Normal Tissue Complication Probability Modeling

For the defined endpoint, two different NTCP modeling approaches were applied: the LKB model, built on generalized equivalent uniform dose (gEUD) (34, 35) and recast for DSHs (23), and the multivariable logistic model. The LKB model parameters (TD_50_, *m* and *n*) and their 95% confidence intervals (CIs) were fitted as described in (36). TD_50_ is the value of the uniform dose given to the entire organ surface corresponding to the 50% probability to induce toxicity; *m* is inversely proportional to the slope of the dose-response curve; and *n* accounts, in this specific case, for the surface effect (*n* close to 0 meaning weak surface effect, *n* close to 1 strong surface effect). Briefly, the Maximum Likelihood method was used to find the best-fit values of the LKB parameters by maximizing the logarithm of the likelihood (LLH). The LLH function was numerically maximized by the Nelder-Mead Simplex Method using an in-house developed library for Matlab. Ninety-five percent confidence intervals for parameters estimates were obtained using the profile likelihood method.

In order to evaluate the possible impact of dosimetric and non-dosimetric factors, the multivariable stepwise logistic regression method for NTCP modeling was also applied (37, 38). In the multivariable analysis were included only the variables highly correlated with RD (*p* < 0.1 at the univariable analysis) that were not collinear (correlation |Rs|<0.75) with variables more correlated with RD.

The Leave-One-Out (LOO) method was applied to the whole statistical pipelines to cross validate the models.

Model performance was evaluated by the area under the receiver operating characteristic (ROC) curve (AUC) and by balanced accuracy (39). Cut-off values on the ROC curve were determined by Youden's J statistic (40). Calibration plots were also generated for graphical assessment of the agreement between observed outcome and LOO prediction.

## Results

**Figure 2:**
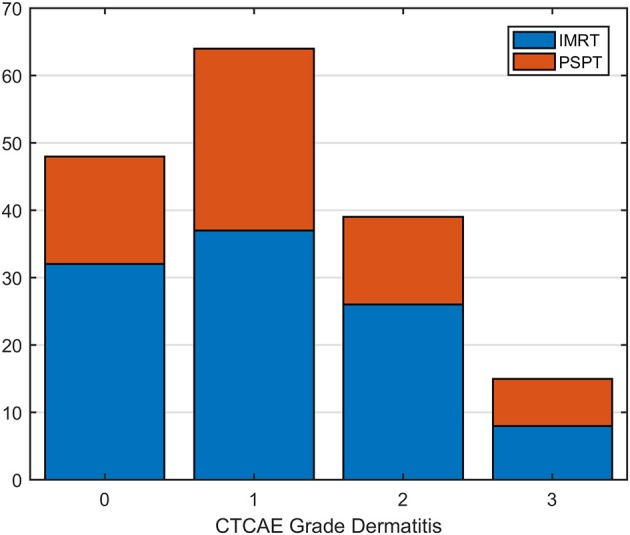
Distribution of radiation dermatitis (RD) grades for patients categorized by treatment modality [Intensity Modulated Radiation Therapy (IMRT) vs. Passive Scattering Proton Therapy (PSPT)].

Of the 166 patients, 118 (71%) developed acute RD of any grade; fifteen of 166 (9%) patients developed G3 RD. In particular, 71 (69%) of IMRT patients developed a RD of any grade compared to 47 (75%) of PSPT patients; G3 RD occurred in 8 IMRT (8%) and 7 (11%) PSPT patients, respectively. The distribution of RD grades for each treatment modality is reported in Figure 2. There were no cases of grade 4 toxicity.

No significant differences were found in the distribution of clinical and disease factors between patients classified according to the treatment modality (Table 1). In addition, the univariate analysis did not show significant correlations between treatment modality and the incidence of RD categorized for any grade threshold (grade ≥ 1: *p* = 0.48; grade ≥ 2: *p* = 0.19; grade ≥ 3: *p* = 0.58).

**Table 1:** Comparison of clinical and disease characteristics between patients classified according to treatment modality.

	**IMRT** **(103 patients)**	**PSPT** **(63 patients)**	*P*-value^*^
**Continuous variables**	**Median (range)**	**Median (range)**	
Age at RT (yr.)	65 (30–85)	67 (39–80)	0.12
GTV Volume (cm^3^)	80.5 (5.8–686.6)	71.0 (1.9–651.8)	0.92
Weight (Kg)	78.2 (48.0–131.4)	81.5 (47.2–122.5)	0.23
Height (cm)	176 (163–180)	176 (164–178)	0.82
BSA (m^2^)	1.95 (1.50–2.43)	1.96 (1.48–5.43)	0.43
**Categorical variables**	**N (%)**	**N (%)**	
**Gender**			0.87
Female	46 (45)	27 (43)	
Male	57 (55)	36 (57)	
**Tumor localization**			0.49
Left lung	32 (33)	24 (40)	
Right lung	65 (67)	35 (60)	
Lower lobe	23 (24)	20 (34)	0.34
Middle lobe	5 (5)	3 (5)	
Upper lobe	69 (71)	35 (58)	
**Prescribed dose**			0.19
66 Gy	44 (43)	20 (32)	
74 Gy	59 (57)	43 (68)	
**Smoking**			0.37
No	10 (10)	3 (5)	
Yes	93 (90)	60 (95)	
**Radiation Dermatitis**			0.6
Grade 0	32 (31)	16 (25)	
Grade 1	37 (36)	27 (43)	
Grade 2	26 (25)	13 (21)	
Grade 3	8 (8)	7 (11)	

*RT, Radiation Therapy; GTV, Gross Tumor Volume; yr., year; BSA, Body Surface Area; IMRT, Intensity Modulated Radiation therapy; PSPT, Passive Scattering Proton Therapy*.

* *Mann–Whitney U test for continuous variables and χ^2^ test for categorical variables*.

**Figure 3:**
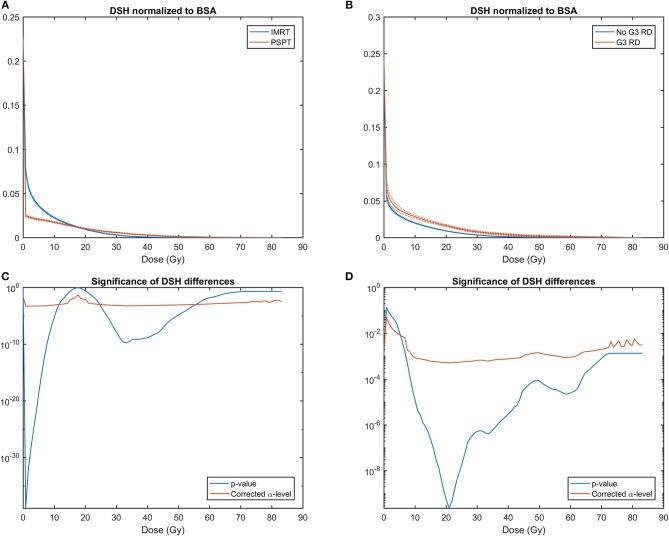
**(A)** Average skin Dose Surface Histograms (DSHs) ± SEM (Standard Error of the Mean) normalized to Body Surface Area (BSA) in patients treated with Intensity Modulated Radiation Therapy (IMRT) and Passive Scattering Proton Therapy (PSPT); **(B)** Average skin DSHs ± SEM normalized to BSA in patients who developed severe (G3) radiation dermatitis (G3-RD) and who did not. SEM are plotted as dashed lines. **(C)** Semi-logarithmic plot for the two-tailed *t*-test between DSH values for PSPT and IMRT at each dose point; **(D)** Semi-logarithmic plot for the two-sample *t*-test between DSH values for G3-RD and unaffected patients. In **(C,D)** blue line for two-tailed *t*-test *p*-value, and red line for α-level of 0.05 corrected for multiple comparison according to Holm–Šidák method.

The analysis of average skin DSH in patients stratified by treatment modality (Figures 3A,C) showed that PSPT, compared to IMRT, significantly reduced the skin surface receiving low doses (<12 Gy). An opposite behavior can be observed in the range from 25 to 55 Gy. Average skin DSHs of patients with and without G3-RD showed instead a significant separation between the two curves starting from the dose value of 5 Gy (Figures 3B,D).

At univariate analysis for patients stratified according to G3 RD (Table 2), all the *S*_*x*_ metrics for doses greater than 5 Gy were significantly correlated with G3-RD; among the clinical variables, total gross tumor volume (GTV) size was the only non dosimetric factor significantly correlated with severe RD (*p* = 0.027).

**Table 2:** Patient, treatment characteristics, dosimetric parameters and univariate analysis against acute grade 3 radiation dermatitis (G3 RD) status.

	**No G3 RD**	**G3 RD**	*P-*value^*^
**Continuous variables**	**Median (range)**	**Median (range)**	
Age at RT (yr)	66 (33–85)	62 (37-74)	0.13
GTV (cm^3^)	71.9 (1.9–686.6)	139.3 (12.2–599.2)	0.03
Weight (Kg)	80 (47–129)	84 (59–131)	0.78
Height (cm)	176 (162–180)	176 (166–180)	0.38
BSA (m^2^)	1.94 (1.48–2.41)	2.02 (1.63–2.43)	0.36
S_5Gy_ (%)	2.8 (0.7–6.0)	3.9 (1.7–6.6)	0.02
S_10Gy_ (%)	1.9 (0.3–3.9)	2.5 (1.5–4.4)	<0.001
S_15Gy_ (%)	1.3 (0.1–2.9)	2.3 (0.1–3.1)	<0.001
S_20Gy_ (%)	0.8 (0.0–2.2)	1.5 (0.7–2.4)	<0.001
S_25Gy_ (%)	0.5 (0.0–2.0)	1.0 (0.3–1.9)	<0.001
S_30Gy_ (%)	0.3 (0.0–1.1)	0.8 (0.3–1.8)	0.001
S_35Gy_ (%)	0.1 (0.0–0.1)	0.5 (0.0–1.7)	<0.001
S_40Gy_ (%)	0.02 (0.00–0.10)	0.3 (0.0–1.6)	<0.001
S_45Gy_ (%)	0.00 (0.00–0.01)	0.01 (0.00–1.50)	<0.001
**Categorical variables**	**N (%)**	**N (%)**	
Gender			0.43
Female	68 (45)	5 (33)	
Male	83 (55)	10 (67)	
Tumor localization			0.40
Left lung	48 (34)	7 (47)	
Right lung	92 (66)	8 (53)	
Lower lobe	39 (28)	4 (27)	0.32
Middle lobe	6 (4)	2 (13)	
Upper lobe	95 (68)	9 (60)	
RT modality			0.58
IMRT	95 (63)	8 (53)	
PSPT	56 (37)	7 (47)	
Smoking			1.00
No	12 (8)	1 (7)	
Yes	139 (92)	14 (93)	

RT, Radiation Therapy; GTV, Gross Tumor Volume; yr., year; BSA, Body Surface Area; IMRT, Intensity Modulated Radiation therapy; PSPT, Passive Scattering Proton Therapy; S_X_ (%), percentage skin surface receiving more than X Gy.

* *Mann–Whitney U-test for continuous variables and χ2 test for categorical variables*.

From NTCP model training, LKB model resulted in the following parameters: TD_50_ = 9.5 Gy (95% CI: [5.9, 18.4] Gy), *m* = 0.24 (95% CI: [0.17, 0.35]), *n* = 0.62 (95% CI: [0.36, 0.92]). Model performance metrics for both training and LOO cross validation were reported in Table 3.

**Table 3:** Normal tissue complication probability (NTCP) model performances for acute grade 3 radiation dermatitis (G3-RD); 95% confidence interval are in brackets.

	**G3-RD NTCP Model**	
**Performance**	**LKB**	**MV Logistic**
AUC	0.82 [0.66, 0.90]	0.85 [0.72, 0.94]
Accuracy	0.67	0.93
Balanced accuracy	0.76	0.78
CV-AUC	0.78 [0.62, 0.88]	0.79 [0.60, 0.90]
CV-Accuracy	0.69	0.91
CV-Balanced accuracy	0.74	0.77
CV-calibration slope (±SE)	0.76 ± 0.19	1.03 ± 0.23
CV-calibration intercept (±SE)	0.008 ± 0.028	−0.003 ± 0.039

*LKB, Lyman-Kutcher-Burman; M, Multivariable; SE, Standard Error; AUC, Area under the ROC curve; CV, cross-validation*.

Regarding the logistic modeling, after the variable selection procedure, multivariable modeling resulted in a single variable model including *S*_20Gy_ (OR = 31.4, 95% CI: [7.5, 131.7], constant = −6.34± 1.03). The ROC analysis identified that the optimal cut-off for *S*_20Gy_ was 1.1% of the BSA.

**Figure 4:**
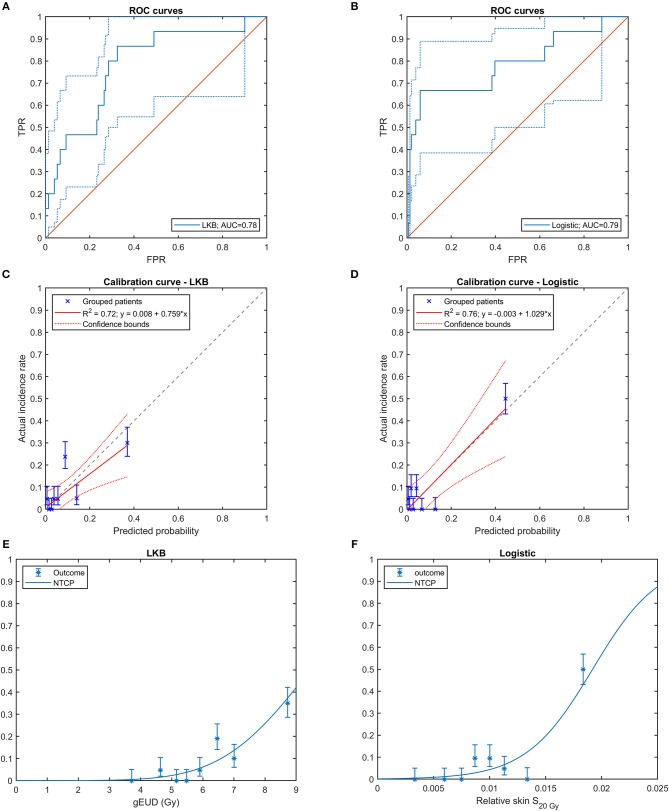
Cross-validated ROC curves of **(A)** Lyman-Kutcher-Burman (LKB) model and **(B)** multivariable logistic regression model (FPR: False Positive Rate, TPR: True Positive Rate); cross-validated calibration plot of **(C)** LKB model and **(D)** logistic model; risk curves with the observed fraction of complications from the data grouped in bins for **(E)** LKB model and **(F)** logistic model. In **(C–F)** the error bars for the reported values represent the 68% confidence intervals.

Similarly, to the LKB model, the logistic model achieved high prediction performances as shown by the AUC values reported in Table 3. LOO cross validation confirmed good prediction and calibration performances (Table 3 and Figure 4). Notably, the balanced accuracy demonstrated a good generalization score and a robust prediction capability despite data imbalance.

## Discussion

The treatment of choice for many thoracic cancers, such as NSCLC, consists in RT given with either concurrent or sequential CHT (10, 41). Radiation induced morbidity to main organs at risk (heart, lungs, esophagus etc.) represents a major concern for radiation treatment. Advanced technologies may potentially reduce the risk of damaging normal tissue, and in particular the favorable physical characteristics of energy deposition in Hadron therapy make it a promising strategy for normal tissue dose sparing and for reducing the side effects of RT.

The skin, however, raises unique issues that deserve a separate discussion. Indeed, the initial dose build-up typical of photons is advantageous for skin sparing, compared to the higher entrance dose deriving from the pile-up of Bragg curves in the production of spread-out Bragg peaks. This effect may lead to an increase in incidence or severity of skin toxicity with a potential detrimental impact on both the RT course and the patient's quality of life. In addition, different amounts of dose may be delivered to the skin depending on the particular technology adopted to give proton therapy, which can rely on either passive scattering or active scanning techniques (42).

The domain of radiation-related skin side effects following proton beam therapy were recently investigated for brain tumor patients (16, 43). Erythema of grade 1-2 was found to be significantly correlated to skin (defined at 3 mm depth) dose volume parameters in the high dose region (*V*_35Gy_) from both passive or active scanning proton beams. In a different study on severe RD following PSPT for breast cancer, the authors identified as prognostic factors the *V*_52.5Gy_ or the *D*_10cc_ of the skin structure defined as a layer of 5 mm inward from the body contour (21).

Few studies have performed a direct comparison on RD incidence following proton versus photon treatments. Acute side effects were compared in a retrospective study on a small cohort of patients after proton beam therapy (18 patients) and IMRT (23 patients) for head and neck cancer (44). Interestingly, in their study, the authors found a greater rate of G2 RD in the proton therapy group, but no difference in the rate of G3 RD between proton and IMRT. Recently, De Cesaris et al. (11) reported on RD after treatment of 86 breast cancer patients undergoing adjuvant proton or photon RT. They observed an increase in moderate (G2) toxicity associated to proton therapy; again, no significant difference between treatment modalities was found for severe RD.

In the present study, we analyzed the data from a randomized trial on PSPT vs. IMRT treatment for inoperable NSCLC patients, and we addressed different aspects related to radiation-induced skin reactions. This study has the unique characteristic of directly comparing acute skin toxicity in a quite large cohort of patients treated at the same institution with proton or photon RT.

First, we analyzed the differences of acute skin toxicity between patients treated with IMRT and PSPT. Both the depth of the lung tumor location within the body and the passive proton technique—used in the trial patients—were expected to increase the skin toxicity of the treatment. Despite this, a key finding of our investigation was that the RT technique did not impact neither incidence nor severity of acute RD (Figure 2).

Then, we evaluated the dose to the skin taking advantage of the DSHs expressly extracted for the epidermis. The DSHs were obtained by a fully automated algorithm that guarantees a high level of standardization. To account for the different patients' sizes, the absolute DSHs were normalized using the BSA (32) as scaling factor. The DSH differences between RT modalities showed that PSPT succeeded in lowering the skin surface receiving low dose (namely, <12 Gy), while the expected increase in entrance dose was evident for intermediate to high dose regime (i.e., higher than 25 Gy). Noteworthy, the switch in dose sparing effectiveness between PSPT and IMRT happens at a dose level in the range from 20 to 30 Gy. This range of doses is known to be strongly related to the probability of radiation-induced dermatological effects (12, 13, 16), as also confirmed in the current study by the comparison of average skin DSHs for patients grouped according to the development of severe RD (Figure 3B).

Since the treatment modality did not correlate with the considered outcome, the NTCP models for severe RD were derived from the whole cohort of patients. We focused on G3 toxicity due to its high clinical relevance. Two different approaches were applied: the traditional purely dosimetric LKB model and the multivariable logistic regression modeling scheme. Both models indeed are important and can find their application in clinical practice. The multivariate logistic model is more flexible when non-dosimetric variables needs to be considered and in order to build predictive tools for improving personalized patient follow-up care. On the other hand, the LKB scheme is more robust for treatment planning optimization (gEUD is a superior evaluator than multiple DSH cut-off points), since it controls the dose distribution over all dose range.

The LKB approach highlighted a relevant surface effect (*n* = 0.62) of the dose on RD development. While the LKB *n* and *m* parameter estimates were comparable with those obtained in previous published models on acute skin toxicity (1, 13), a TD_50_ of 10 Gy was a relatively low dose when compared to those previous studies. However, a direct comparison was hampered by the different modeling strategy (LKB recast on DSH) or the different normalization procedure (the BSA as scaling factor) adopted in the present analysis.

On the other hand, the multivariable logistic regression model highlighted that the most and only significantly independent toxicity predictor was the skin surface receiving more than 20 Gy. The robustness of those radiobiological hints is supported by the good performances of both predictive models, which showed cross-validated ROC-AUCs close to 0.8.

The current interest in the investigation on the patterns of dose-RD response is enhanced by the increasing attention to the quality of life of patients undergoing RT, in turn triggered by the substantially improved therapeutic ratio of the modern treatment techniques. Precise knowledge of the radiobiology of acute skin radiation effects constitutes the essential basis for the development of biology-based treatment strategies. In addition, severe acute skin reactions may be prodromal of consequential skin late effects (45), thus making their prediction and, possibly, prevention even more important.

The newest proton facilities have moved toward pencil beam scanning technology. A phantom dosimetric study investigating skin dose differences between spot scanning and passively scattered proton therapy beams indicated that, on average, a lower skin dose of about 12% was delivered when active spot scanning proton beams were used (42). Thanks to the higher flexibility with an enhanced modulation capability, the combined use of active scanning beams and the inclusion of skin specific model parameters in the planning strategies may result in further skin dose sparing to minimize the occurrence of cutaneous toxicity. In this respect, we focused on two classes of NTCP models that could be easily ported on the most common treatment planning systems used in the clinical practice. Indeed, the DSH formalism can be implemented following the procedure suggested in (23), thus directly allowing for the application of the dose constraints (e.g., *S*_20Gy_) derived by the logistic approach. On the other side, the estimation of the *n* parameter of the LKB strategy can be exploited for treatment plan optimization by constraining the gEUD, which is a widespread empirical model available in several commercial systems.

In order to improve our understanding of the mechanisms underlying radiation-induced skin damage, future direction of the research is the inclusion of spatial information of dose distributions within the analysis of skin toxicity, as already performed for different toxicity endpoints after RT (46–50). The extraction of organ Dose-Surface Maps (51, 52) may allow for an enhanced prediction of RT toxicity based on the knowledge of the most radiosensitive skin areas.

Additional issues to be considered when modeling RD should be the impact of CHT treatments and of different RT dose fractionation schemes. Radiation-related skin side effects have been associated to different patient-related factors such as the use of radiosensitizing CHT and/or biologics (1). In particular, both incidence and severity of RD may be increased by concomitant CHT, although conflicting results are reported in the available literature. For example, a randomized comparison of patients treated for anal cancer by RT alone or combined with CHT found overall RD in 76% for radiation alone versus 93% for combined modality therapy (53). In contrast, a three-arm randomized trial in advanced larynx cancer found similar Grade 3–4 acute skin toxicities for patients receiving RT alone (9%), concurrent RT-CHT (10%), and sequential CHT-RT (7%) (54). Rates of acute and late skin toxicity were not significantly different also in a retrospective analysis of breast cancer patients undergoing lumpectomy with or without adjuvant CHT followed by hypofractionated RT (55). Recently, a multivariable NTCP analysis did not highlighted any effect of CHT on severe RD in breast cancer patients (13).

As regards to dose fractionation, greater dose per fraction are generally of concern to normal tissue toxicities. However, data on adverse skin reactions on patients who underwent Stereotactic Body Radiation Therapy (SBRT) is still limited (1). Suggested skin SBRT dose constraints (for toxicity grade ≥ 3) were D_10cc_ <23 Gy, for one single fraction of 34 Gy, and D_10cc_ <30-33 Gy for a total dose of 40-60 Gy in 4-5 fractions (56). Interestingly, these dose constraints are in the range of doses strongly related to the probability of RD (Figure 3B).

In the cohort analyzed in the current study, all patients received concurrent CHT and standard fractionation regimens. Future studies on large cohorts of patients undergoing RT with and without the use of CHT treatments and with different fractionation size are warranted in order to shed light on the possible CHT enhancement factor and fractionation effects.

A potential limitation of the study is related to the dose calculation uncertainties in the first few millimeters from body surface, which may be relatively large. However, in order to quantify their impact on the modeling results, Mori et al. (15) performed a sensitivity analysis showing that dose uncertainty has negligible impact on logistic regressions coefficients. Furthermore, the percentage differences between the measured dose to the skin and the estimate of the treatment planning system with passively scattered proton beams was evaluated in (42). The average measured doses resulted to be only 2% lower than the average calculated doses.

In conclusion, despite skin toxicity has long been considered a potential limiting factor in the clinical use of proton beam therapy, no significant differences in RD incidence was found between IMRT and PSPT in the analyzed trial. The developed NTCP models for the prediction of severe RD, once externally validated, may advance treatment planning optimization for the implementation of skin sparing techniques.

## Data Availability Statement

The datasets for this article are not publicly available because: Data Sharing Agreement does not include this option. Requests to access the datasets should be directed to Dr. Zhongxing Liao (zliao@mdanderson.org).

## Ethics Statement

The studies involving human participants were reviewed and approved by MD Anderson Cancer Center IRB (protocol NCT00915005). The patients/participants provided their written informed consent to participate in this study.

## Author Contributions

GP and LC conceived and designed the study. ZL, TX, SH, and RM participated in patient recruitment and collected the clinical data. GP, SM, MC, and LC processed the data. GP and SM analyzed the results. GP and LC wrote the manuscript. All authors reviewed the final manuscript.

### Conflict of Interest

The authors declare that the research was conducted in the absence of any commercial or financial relationships that could be construed as a potential conflict of interest.

## References

[1] Avanzo M Stancanello J Jena R Adverse effects to the skin and subcutaneous tissue. In: Rancati T Fiorino C, editors. Modelling Radiotherapy Side Effects: Practical Applications For Planning Optimisation CRC Press (2019). 10.1201/b21956-12

[2] Hymes SR Strom EA Fife C. Radiation dermatitis: clinical presentation, pathophysiology, and treatment 2006. J Am Acad Dermatol. (2006) 54:28–46. 10.1016/j.jaad.2005.08.054 16384753

[3] Decker RH Strom EA Wilson LD Skin surface, dermis, and wound healing. In: Rubin P Constine LS Marks LB, editors. ALERT-Adverse Late Effects of Cancer Treatment: Volume 2. Normal Tissue Specific Sites and Systems. Springer (2013). p. 189 10.1007/978-3-540-75863-1_9

[4] Jiang ZQ Yang K Komaki R Wei X Tucker SL Zhuang Y. Long-term clinical outcome of intensity-modulated radiotherapy for inoperable non-small cell lung cancer: the MD Anderson experience. Int J Radiat Oncol Biol Phys. (2012) 83:332–9. 10.1016/j.ijrobp.2011.06.1963 22079735

[5] Palm A Johansson KA. A review of the impact of photon and proton external beam radiotherapy treatment modalities on the dose distribution in field and out-of-field; implications for the long-term morbidity of cancer survivors. Acta Oncol. (2007) 46:462–73. 10.1080/02841860701218626 17497313

[6] Pacelli R Caroprese M Palma G Oliviero C Clemente S Cella L. Technological evolution of radiation treatment: Implications for clinical applications. Semin Oncol. (2019) 46:193–201. 10.1053/j.seminoncol.2019.07.004 31395286

[7] Freedman GM Anderson PR Li J Eisenberg DF Hanlon AL Wang L. Intensity modulated radiation therapy (IMRT) decreases acute skin toxicity for women receiving radiation for breast cancer. Am J Clin Oncol. (2006) 29:66–70. 10.1097/01.coc.0000197661.09628.03 16462506

[8] Pignol JP Olivotto I Rakovitch E Gardner S Sixel K Beckham W. A multicenter randomized trial of breast intensity-modulated radiation therapy to reduce acute radiation dermatitis. J Clin Oncol. (2008) 26:2085–92. 10.1200/JCO.2007.15.2488 18285602

[9] Cella L Lomax A Miralbell R. New techniques in hadrontherapy: intensity modulated proton beams. Phys Med. (2001) 17(Suppl. 1):100–2. 11770521

[10] Brooks ED Ning MS Verma V Zhu XR Chang JY Proton therapy for non-small cell lung cancer: the road ahead. Trans Lung Cancer Res. (2019) 2019:S202–S12. 10.21037/tlcr.2019.07.08 PMC6795573 31673525

[11] DeCesaris CM Rice SR Bentzen SM Jatczak J Mishra MV Nichols EM. Quantification of acute skin toxicities in patients with breast cancer undergoing adjuvant proton versus photon radiation therapy: a single institutional experience. Int J Radiat Oncol Biol Phys. (2019) 104:1084–90. 10.1016/j.ijrobp.2019.04.015 31028831

[12] Turesson I Thames HD. Repair capacity and kinetics of human skin during fractionated radiotherapy: erythema, desquamation, and telangiectasia after 3 and 5 year's follow-up. Radiother Oncol. (1989) 15:169–88. 10.1016/0167-8140(89)90131-X 2762590

[13] Pastore F Conson M D'Avino V Palma G Liuzzi R Solla R. Dose-surface analysis for prediction of severe acute radio-induced skin toxicity in breast cancer patients. Acta Oncol. (2016) 55:466–73. 10.3109/0284186X.2015.1110253 26623532

[14] Parekh A Dholakia AD Zabranksy DJ Asrari F Camp M Habibi M. Predictors of radiation-induced acute skin toxicity in breast cancer at a single institution: role of fractionation and treatment volume. Adv Radiat Oncol. (2018) 3:8–15. 10.1016/j.adro.2017.10.007 29556573 PMC5856985

[15] Mori M Cattaneo GM Dell'Oca I Foti S Calandrino R Di Muzio NG. Skin DVHs predict cutaneous toxicity in Head and Neck Cancer patients treated with Tomotherapy. Phys Med. (2019) 59:133–41. 10.1016/j.ejmp.2019.02.015 30824367

[16] Dutz A Luhr A Agolli L Troost EGC Krause M Baumann M. Development and validation of NTCP models for acute side-effects resulting from proton beam therapy of brain tumours. Radiother Oncol. (2019) 130:164–71. 10.1016/j.radonc.2018.06.036 30033385

[17] Alexander MA Brooks WA Blake SW Normal tissue complication probability modeling of tissue fibrosis following breast radiotherapy. Phys Med Biol. (2007) 52:1831–43. 10.1088/0031-9155/52/7/005 17374914

[18] Avanzo M Stancanello J Trovo M Jena R Roncadin M Trovo MG. Complication probability model for subcutaneous fibrosis based on published data of partial and whole breast irradiation. Phys Med. (2012) 28:296–306. 10.1016/j.ejmp.2011.11.002 22119271

[19] Mukesh MB Harris E Collette S Coles CE Bartelink H Wilkinson J. Normal tissue complication probability (NTCP) parameters for breast fibrosis: pooled results from two randomised trials. Radiother Oncol. (2013) 108:293–8. 10.1016/j.radonc.2013.07.006 23953408

[20] Kindts I Defraene G Laenen A Petillion S Van Limbergen E Depuydt T. Development of a normal tissue complication probability model for late unfavourable aesthetic outcome after breast-conserving therapy. Acta Oncol. (2018) 57:916–23. 10.1080/0284186X.2018.1461926 29652212

[21] Liang X Bradley JA Zheng D Rutenberg M Yeung D Mendenhall N. Prognostic factors of radiation dermatitis following passive-scattering proton therapy for breast cancer. Radiat Oncol. (2018) 13:72. 10.1186/s13014-018-1004-3 29673384 PMC5909216

[22] Borm KJ Loos M Oechsner M Mayinger MC Paepke D Kiechle MB. Acute radiodermatitis in modern adjuvant 3D conformal radiotherapy for breast cancer - the impact of dose distribution and patient related factors. Radiat Oncol. (2018) 13:218. 10.1186/s13014-018-1160-5 30404664 PMC6223003

[23] Palma G Cella L. A new formalism of dose surface histograms for robust modeling of skin toxicity in radiation therapy. Phys Med. (2019) 59:75–8. 10.1016/j.ejmp.2019.02.005 30928068

[24] Zhang X Li Y Pan X Xiaoqiang L Mohan R Komaki R. Intensity-modulated proton therapy reduces the dose to normal tissue compared with intensity-modulated radiation therapy or passive scattering proton therapy and enables individualized radical radiotherapy for extensive stage IIIB non-small-cell lung cancer: a virtual clinical study. Int J Radiat Oncol Biol Phys. (2010) 77:357–66. 10.1016/j.ijrobp.2009.04.028 19660879 PMC2868090

[25] Hui Z Zhang X Starkschall G Li Y Mohan R Komaki R. Effects of interfractional motion and anatomic changes on proton therapy dose distribution in lung cancer. Int J Radiat Oncol Biol Phys. (2008) 72:1385–95. 10.1016/j.ijrobp.2008.03.007 18486357 PMC3401022

[26] Murshed H Liu HH Liao Z Barker JL Wang X Tucker SL. Dose and volume reduction for normal lung using intensity-modulated radiotherapy for advanced-stage non-small-cell lung cancer. Int J Radiat Oncol Biol Phys. (2004) 58:1258–67. 10.1016/j.ijrobp.2003.09.086 15001271

[27] Liao Z Lee JJ Komaki R Gomez DR O'Reilly MS Fossella FV. Bayesian adaptive randomization trial of passive scattering proton therapy and intensity-modulated photon radiotherapy for locally advanced non-small-cell lung cancer. J Clin Oncol. (2018) 36:1813–22. 10.1200/JCO.2017.74.0720 29293386 PMC6008104

[28] Palma G Monti S Xu T Scifoni E Yang P Hahn SM. Spatial dose patterns associated with radiation pneumonitis in a randomized trial comparing intensity-modulated photon therapy with passive scattering proton therapy for locally advanced non-small cell lung cancer. Int J Radiat Oncol Biol Phys. (2019) 104:1124–32. 10.1016/j.ijrobp.2019.02.039 30822531

[29] Deasy JO Blanco AI Clark VH. CERR: a computational environment for radiotherapy research. Med Phys. (2003) 30:979–85. 10.1118/1.1568978 12773007

[30] Archambeau JO Pezner R Wasserman T. Pathophysiology of irradiated skin and breast. Int J Rad Oncol. (1995) 31:1171–85. 10.1016/0360-3016(94)00423-I 7713781

[31] Shih FY Image Processing and Mathematical Morphology: Fundamentals and Applications. CRC Press (2017). 10.1201/9781420089448

[32] Du Bois D Du Bois EF Clinical calorimetry: tenth paper a formula to estimate the approximate surface area if height and weight be known. Arch Inter Med. (1916) 17:863–71. 10.1001/archinte.1916.00080130010002

[33] Holm S A simple sequentially rejective multiple test procedure. Scand J Stat. (1979) 1979:65–70.

[34] Luxton G Hancock SL Boyer AL. Dosimetry and radiobiologic model comparison of IMRT and 3D conformal radiotherapy in treatment of carcinoma of the prostate. Int J Rad Oncol. (2004) 59:267–84. 10.1016/j.ijrobp.2004.01.024 15093924

[35] Niemierko A A generalized concept of equivalent uniform dose (EUD). Med Phys. (1999) 1999:26. 10.1118/1.598063 9029544

[36] Cella L Palma G Deasy JO Oh JH Liuzzi R D'Avino V. Complication probability models for radiation-induced heart valvular dysfunction: do heart-lung interactions play a role? PLoS ONE. (2014) 9:e111753. 10.1371/journal.pone.0111753 25360627 PMC4216137

[37] Cella L D'Avino V Liuzzi R Conson M Doria F Faiella A. Multivariate normal tissue complication probability modeling of gastrointestinal toxicity after external beam radiotherapy for localized prostate cancer. Radiat Oncol. (2013) 8:221. 10.1186/1748-717X-8-221 24053357 PMC3852304

[38] Cella L Liuzzi R Conson M D'Avino V Salvatore M Pacelli R. Multivariate normal tissue complication probability modeling of heart valve dysfunction in Hodgkin lymphoma survivors. Int J Radiat Oncol Biol Phys. (2013) 87:304–10. 10.1016/j.ijrobp.2013.05.049 23886419

[39] Brodersen KH Ong CS Stephan KE Buhmann JM editors. The balanced accuracy and its posterior distribution. In: 2010 20th International Conference on Pattern Recognition. (2010). 10.1109/ICPR.2010.764

[40] Youden WJ. Index for rating diagnostic tests. Cancer. (1950) 3:32–5. 10.1002/1097-0142(1950)3:1<32::AID-CNCR2820030106>3.0.CO;2-3 15405679

[41] Liao Z Simone CB 2nd. Particle therapy in non-small cell lung cancer. Transl Lung Cancer Res. (2018) 7:141–52. 10.21037/tlcr.2018.04.11 29876313 PMC5960664

[42] Arjomandy B Sahoo N Cox J Lee A Gillin M. Comparison of surface doses from spot scanning and passively scattered proton therapy beams. Phys Med Biol. (2009) 54:N295–302. 10.1088/0031-9155/54/14/N02 19550003

[43] Palma G Taffelli A Fellin F D'Avino V Scartoni D Tommasino F. Modelling the risk of radiation induced alopecia in brain tumor patients treated with scanned proton beams. Radiother Oncol. (2019) 144:127–34. 10.1016/j.radonc.2019.11.013 31805517

[44] Romesser PB Cahlon O Scher E Zhou Y Berry SL Rybkin A. Proton beam radiation therapy results in significantly reduced toxicity compared with intensity-modulated radiation therapy for head and neck tumors that require ipsilateral radiation. Radiother Oncol. (2016) 118:286–92. 10.1016/j.radonc.2015.12.008 26867969 PMC4980117

[45] Dorr W Hendry JH. Consequential late effects in normal tissues. Radiother Oncol. (2001) 61:223–31. 10.1016/S0167-8140(01)00429-7 11730991

[46] Monti S Palma G D'Avino V Gerardi M Marvaso G Ciardo D. Voxel-based analysis unveils regional dose differences associated with radiation-induced morbidity in head and neck cancer patients. Sci Rep. (2017) 7:7220. 10.1038/s41598-017-07586-x 28775281 PMC5543173

[47] Mylona E Acosta O Lizee T Lafond C Crehange G Magne N. Voxel-based analysis for identification of urethrovesical subregions predicting urinary toxicity after prostate cancer radiation therapy. Int J Radiat Oncol Biol Phys. (2019)2019:88. 10.1016/j.ijrobp.2019.01.088 30716523

[48] Palma G Monti S Buonanno A Pacelli R Cella L. PACE: a probabilistic atlas for normal tissue complication estimation in radiation oncology. Front Oncol. (2019) 9:130. 10.3389/fonc.2019.00130 30918837 PMC6424876

[49] Palma G Monti S D'Avino V Conson M Liuzzi R Pressello MC. A voxel-based approach to explore local dose differences associated with radiation-induced lung damage. Int J Radiat Oncol Biol Phys. (2016) 96:127–33. 10.1016/j.ijrobp.2016.04.033 27511851 PMC5533285

[50] Yahya N Ebert MA House MJ Kennedy A Matthews J Joseph DJ. Modeling urinary dysfunction after external beam radiation therapy of the prostate using bladder dose-surface maps: evidence of spatially variable response of the bladder surface. Int J Radiat Oncol Biol Phys. (2017) 97:420–6. 10.1016/j.ijrobp.2016.10.024 28068247

[51] Buettner F Gulliford SL Webb S Partridge M. Using dose-surface maps to predict radiation-induced rectal bleeding: a neural network approach. Phys Med Biol. (2009) 54:5139–53. 10.1088/0031-9155/54/17/005 19661568

[52] Dankers F Wijsman R Troost EG Monshouwer R Bussink J Hoffmann AL Esophageal wall dose-surface maps do not improve the predictive performance of a multivariable NTCP model for acute esophageal toxicity in advanced stage NSCLC patients treated with intensity-modulated (chemo-)radiotherapy. Phys Med Biol. (2017) 62:3668–81. 10.1088/1361-6560/aa5e9e 28379845

[53] Party UACTW Epidermoid anal cancer: results from the UKCCCR randomised trial of radiotherapy alone versus radiotherapy, 5-fluorouracil, and mitomycin. The Lancet. (1996) 348:1049–54. 10.1016/S0140-6736(96)03409-5 8874455

[54] Forastiere AA Goepfert H Maor M Pajak TF Weber R Morrison W. Concurrent chemotherapy and radiotherapy for organ preservation in advanced laryngeal cancer. N Engl J Med. (2003) 349:2091–8. 10.1056/NEJMoa031317 14645636

[55] Hijal T Al Hamad AA Niazi T Sultanem K Bahoric B Vuong T Hypofractionated radiotherapy and adjuvant chemotherapy do not increase radiation-induced dermatitis in breast cancer patients. Curr Oncol. (2010) 17:22–7. 10.3747/co.v17i5.604 20975875 PMC2949365

[56] Benedict SH Yenice KM Followill D Galvin JM Hinson W Kavanagh B. Stereotactic body radiation therapy: the report of AAPM Task Group 101. Med Phys. (2010) 37:4078–101. 10.1118/1.3438081 20879569

